# Productivity, Disturbance and Ecosystem Size Have No Influence on Food Chain Length in Seasonally Connected Rivers

**DOI:** 10.1371/journal.pone.0066240

**Published:** 2013-06-12

**Authors:** Danielle M. Warfe, Timothy D. Jardine, Neil E. Pettit, Stephen K. Hamilton, Bradley J. Pusey, Stuart E. Bunn, Peter M. Davies, Michael M. Douglas

**Affiliations:** 1 Research Institute for the Environment and Livelihoods, Charles Darwin University, Darwin, Northern Territory, Australia; 2 Toxicology Centre, University of Saskatchewan, Saskatoon, Saskatchewan, Canada; 3 Australian Rivers Institute, Griffith University, Nathan, Queensland, Australia; 4 Centre of Excellence in Natural Resource Management, The University of Western Australia, Albany, Western Australia, Australia; 5 Kellogg Biological Station and Department of Zoology, Michigan State University, Hickory Corners, Michigan, United States of America; CNRS, University of Montpellier II, France

## Abstract

The food web is one of the oldest and most central organising concepts in ecology and for decades, food chain length has been hypothesised to be controlled by productivity, disturbance, and/or ecosystem size; each of which may be mediated by the functional trophic role of the top predator. We characterised aquatic food webs using carbon and nitrogen stable isotopes from 66 river and floodplain sites across the wet-dry tropics of northern Australia to determine the relative importance of productivity (indicated by nutrient concentrations), disturbance (indicated by hydrological isolation) and ecosystem size, and how they may be affected by food web architecture. We show that variation in food chain length was unrelated to these classic environmental determinants, and unrelated to the trophic role of the top predator. This finding is a striking exception to the literature and is the first published example of food chain length being unaffected by any of these determinants. We suggest the distinctive seasonal hydrology of northern Australia allows the movement of fish predators, linking isolated food webs and potentially creating a regional food web that overrides local effects of productivity, disturbance and ecosystem size. This finding supports ecological theory suggesting that mobile consumers promote more stable food webs. It also illustrates how food webs, and energy transfer, may function in the absence of the human modifications to landscape hydrological connectivity that are ubiquitous in more populated regions.

## Introduction

The food web is a central organizing theme in ecology, depicting the feeding relationships between species in a community [1], [2] and providing a framework for understanding energy transfer and biogeochemical processes [3], biodiversity and trophic interactions [4], consumer behaviour and movement [5], [6], and community stability and persistence in the face of perturbation [2], [7], [8]. Food web structure is often summarised by emergent properties such as food chain length (FCL), which measures the number of energy transfers between the base and the top of a food web, and is considered a central attribute of ecological communities [9]. Food chain length influences structural attributes of communities such as species diversity, trophic interactions and predator abundance [10], [11], as well as functional attributes such as population stability, primary and secondary production, material cycling, and contaminant bioaccumulation [1], [12]–[14].

Variation in FCL has long been observed in natural communities [1] and is hypothesised to be controlled by basal productivity, disturbance and/or ecosystem size [13], [15]. The productivity or resource availability hypothesis states that because energy is lost through each successive transfer up the food chain, FCL is limited by available energy resources [16]. The disturbance hypothesis predicts shorter food chains in more disturbed ecosystems due to either longer food chains being less resilient to perturbations than shorter food chains [17], or species at higher trophic levels being rarer and more likely to be lost during disturbance events [18], [19]. The ecosystem size hypothesis [20] predicts that larger ecosystems will have longer food chains because they support greater species richness [21], support more basal resources [22], promote coexistence of predators and prey [15], [23], promote population persistence through enhanced colonisation opportunity [11], [23], and/or support greater functional trophic diversity and less omnivory [20].

Despite having been proposed decades ago, the empirical support for any one of these environmental determinants being a dominant influence on FCL remains equivocal; rather, it is more likely that multiple factors control FCL [11], [13], [24]. Productivity has been shown to have either neutral or positive effects on FCL, disturbance tends to limit FCL, and ecosystem size generally lengthens food chains (Table 1). A recent meta-analysis of the 13 field studies that tested one or more determinants (using the correlation coefficient as an index of effect size) found that productivity and ecosystem size both positively influenced FCL, whereas disturbance did not significantly shorten food chains [25]. Intriguingly, this meta-analysis also showed that although productivity generally increased FCL, the magnitude of ecosystem size and disturbance effects were highly variable and could include positive, neutral and negative effects on FCL [25]. Only two studies, both in temperate riverine ecosystems, have tested all three environmental determinants concurrently: both found FCL was not affected by productivity, but increased with ecosystem size and decreased with disturbance [26], [27] (Table 1). These studies showed that either larger ecosystems attenuate the effects of disturbance, thereby enhancing environmental stability and supporting longer food chains [26], or concluded that effects of disturbance on productivity are exacerbated in smaller systems leading to increased omnivory and shorter food chains [27].

**Table 1:** Summary of findings from studies which have concurrently tested one or more environmental determinants of food chain length.

Study	Ecosystem type	Sample size	Environmental determinant			Determinants independent?
			productivity	ecosystem size	disturbance	
Pimm and Kitching 1987 [19]	Artificial treeholes	3	0		−	yes
Jenkins et al. 1992 [18]	Artificial treeholes	15	+		−	yes
Warren and Spencer 1996 [78]	Pond mesocosms	4	0		0	yes
Spencer and Warren 1996 [79]	Laboratory microcosms	12	0	+		yes
Schneider 1997 [80]	Temperate ponds	7			−	
Kaunzinger and Morin 1998 [81]	Laboratory microcosms	12	+			
Townsend et al. 1998 [68]	Temperate streams	10	+		0	yes
Vander Zanden et al. 1999 [29]	Temperate lakes	14	0	+		no
Post et al. 2000 [20]	Temperate lakes	25	0	+		yes
Jennings and Warr 2003 [82]	Marine	74	0		−	
Thompson and Townsend 2005 [24]	Temperate streams	18	+	0		yes
Williams and Trexler 2006 [83]	Tropical wetlands	20	0		−	yes
Hoeinghaus et al. 2008 [44]	Tropical rivers & reservoirs	10	+			
Stenroth et al. 2008 [84]	Temperate lakes	18	+	0		yes
Takimoto et al. 2008 [85]	Tropical islands	36		+	0	yes
Walters and Post 2008 [67]	Temperate streams	6			0	
Doi et al. 2009 [66]	Temperate ponds	15	+	+		yes
McHugh et al. 2010 [27]	Temperate streams	16	0	+	−	no
Sabo et al. 2010 [26]	Temperate rivers	36	0	+	−	no
Reid et al. 2012 [86]	Temperate billabongs	10		+		

+ indicates significant positive effect on FCL.

− indicates significant negative effect on FCL.

0 indicates non-significant effect.

Absence of symbol indicates the determinant was not tested.

Such variable findings, and conclusions, are likely due to the fact that FCL is an aggregate property of food webs, reflecting changes in food web structure that can be generated by multiple mechanisms [4], [28]. Food chain length can be altered by the addition or removal of a top consumer (additive mechanism), the addition or removal of an intermediate consumer (insertion), or a change in the degree of trophic omnivory shown by a top consumer (omnivory) [28], [29]. In particular, the degree of omnivory or the strength of intraguild predation displayed by a top predator has been theoretically shown to mediate the influence of the above-mentioned environmental determinants, limiting FCL under increasing productivity or reduced disturbance but increasing FCL in larger ecosystems [15]. Therefore, examining the trophic role of top predators concurrently with FCL responses to environmental determinants is likely to be instructive in understanding the mechanisms by which these determinants control FCL.

We used carbon and nitrogen stable isotopes to assess the influence of productivity, disturbance and ecosystem size on food chain length, as well as the trophic role of the top predator, in river-floodplain ecosystems of the wet-dry tropics in northern Australia. The strongly seasonal, wet-dry climate and the relatively unimpeded flow regimes of this region [30] give rise to spatiotemporal gradients in hydrological connection and isolation [31], [32]. This regime of hydrological connectivity can influence patterns in biotic assemblage composition [33], in the strength of coupling between consumers and their local resources [34], in local environmental conditions affecting ecosystem structure [35], [36], and in the movement of top predators [6], [37]. Together, these patterns suggest that food web structure, and hence food chain length, should vary according to local environmental conditions and provide an opportunity to investigate the mechanisms underpinning such variation. Accordingly, we predicted that 1) more productive sites, as indicated by nutrient concentrations, would have longer food chains, 2) more hydrologically isolated sites, which serve as an analog for more disturbed sites in this landscape setting, would have shorter food chains, and 3) larger ecosystems would have longer food chains. We also predicted that the strength of these relationships would be related to degree of trophic omnivory in the top predator, where food webs with omnivores (i.e. intraguild predators) rather than piscivores as the top predator would have shorter food chains but would still show a positive relationship between FCL and ecosystem size [15].

We show that in fact, none of these classic determinants have any influence on FCL in our seasonally-connected rivers, nor is FCL related to the trophic role of the top predator. Our finding is a striking exception to the literature and well-established patterns in food web ecology [25] (Table 1), and illustrates how food webs, and thus energy transfer, may be structured in the absence of human modifications that disrupt hydrological connectivity across landscapes.

## Materials and Methods

### Ethics Statement

All field sampling and collection of tissue samples was conducted under animal ethics permits from Charles Darwin University (A08008), Griffith University (ENV/08/08/AEC) and The University of Western Australia (RA/3/100/765), faunal sampling permits from the Northern Territory (DPIF S17/2666), Queensland (DAFF 89212) and Western Australian (DEC SF0063279, DOF 2008-46) Governments, and research permits from the Northern and Kimberley Land Councils to work on Aboriginal land. The giant freshwater whipray (*Himantura chaophraya*) and the freshwater sawfish (*Pristis microdon*), both threatened under the Australian Government's EPBC Act and on the IUCN Red List, were very occasionally sampled during electrofishing but returned to the water unharmed.

### Study area

**Figure 1:**
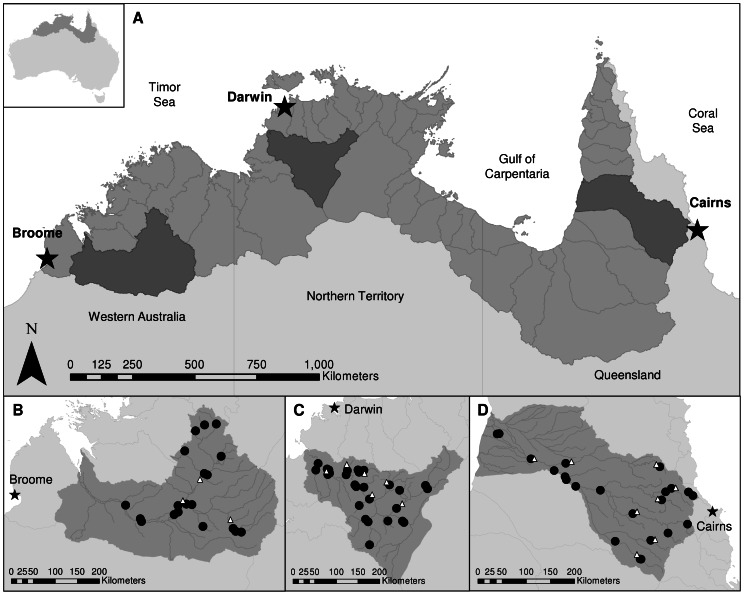
Map of the study area and sample sites. A) The region of the wet-dry tropics of northern Australia (mid-grey), with study catchments highlighted (dark grey). States and territories are labelled, as are major towns (stars) and gauging stations (white triangles). B) The Fitzroy River catchment (96,000 km^2^, n = 18 sites), C) the Daly River catchment (55,000 km^2^, n = 26 sites), and D) the Mitchell River catchment (72,000 km^2^, n = 22 sites) showing sampling locations (black circles).

The wet-dry tropics of northern Australia cover approximately one fifth of the continent's land-area (about 1.3 million km^2^; Fig. 1). The region is generally of low topographical relief (under 550 m altitude) and dominated by grassy woodland savanna that supports a large cattle grazing industry. Population density is very low (1 person per 2.5 km^2^), with approximately 90,000 people in the largest urban centre, Darwin. Consequently, infrastructure is minimal and many of these river systems are remote and inaccessible, largely ungauged, and among the least impacted in the country [30] and the world [38]. Annual rainfall varies from 300–600 mm along the southern boundary of the region, increasing to up to 1000–2000 mm along the coast (Bureau of Meteorology, www.bom.gov.au), with most falling predictably during the summer monsoon season from October to April. Peak discharges in rivers during the wet season can be large but show high inter-annual variation [32]. Lowland floodplains can represent up to a third of the catchment area [39] and while vast areas can be inundated, the duration of inundation can be highly variable, lasting from days to weeks and, in a few catchments, months [34]. As rainfall ceases, many rivers across the region recede to a series of disconnected waterholes during the winter dry season. Hydrological classifications of rivers across northern Australia characterise them as either perennial, which are groundwater-fed and relatively uncommon, seasonally intermittent with flow ceasing for the dry season (the most common river type), or extremely intermittent, which only flow for short periods during the wet season [32], [40].

We collected carbon and nitrogen stable isotope data on food webs from 66 sites across three catchments during the 2008 dry season: 26 sites in the Daly River catchment (Northern Territory), 22 sites in the Mitchell River catchment (Queensland), and 18 sites in the Fitzroy River catchment (Western Australia; Fig. 1). Sites ranged from 13–18° S latitude and 124–145°E longitude, and were stratified according to whether they occurred on main river channels, floodplain waterholes or tributaries (Fig. S1), the latter being more common across this landscape. Sites were selected to cover gradients in productivity, disturbance and ecosystem size and on the basis of accessibility, so were representative rather than random. Post-hoc power analysis showed that sampling 18 sites (the minimum number of sites within a catchment) was sufficient to detect an effect size of *r* = 0.60 at α = 0.05 significance, providing 0.86 power of not making a Type II error. This effect size was based on the largest average effect size in a meta-analysis on the effects of productivity (*r* = 0.50), disturbance (*r* = −0.28) and ecosystem size (*r* = 0.60) on FCL [25].

### Food web sampling

Potential sources and consumers were sampled from multiple locations across each site to encompass the range of habitats present and obtain as representative a food web as possible (full sampling details are provided in Jardine et al. [34]). Primary sources included plant material from within and outside the water. Whole samples of conditioned leaf litter (cleaned of biofilm), grasses and emergent and floating-leaved macrophytes were collected to represent terrestrial production because they obtain CO_2_ from the air, while aquatic sources included submerged macrophytes, charophytes, filamentous algae and biofilm. Biofilm was scrubbed from submerged surfaces (rocks, wood and/or macrophytes), and left undisturbed in a 1 L measuring cylinder for 20 min to allow sediments and detritus to settle out, leaving the top greenish fraction that we extracted and filtered [34]. Consumers included zooplankton, macroinvertebrates, crustaceans and fish. Zooplankton were collected from main channel and waterhole sites by conducting sub-surface tows with a 150 µm net, and benthic and epiphytic macroinvertebrates were sampled using a combination of dip-netting, kick-sampling and baited traps. Macroinvertebrates were live-picked and identified to Family, and enough material collected from across the site to obtain multiple samples for each Family present. Fish and larger crustaceans were collected using both backpack and boat-mounted electrofishing units, for at least 50 min fishing time and intentionally targeting the full range of habitats present. At least three individuals of each fish species, covering the range of body sizes sampled, were kept for white muscle tissue samples from the dorsal muscle, although occasionally non-lethal clips of the anal fins (with isotope ratios that are strongly correlated with muscle tissue [41]) were collected from large individuals. All samples were kept on ice or frozen for transport back to the laboratory and were prepared and analysed for their carbon and nitrogen stable isotopes as described in Jardine et al. [34]. All stable isotope data and environmental data collected from each site are available from the publicly-accessible Tropical Rivers and Coastal Knowledge (TRaCK) Digital Atlas website (http://atlas.track.org.au/).

Food chain length was defined as maximum trophic position (TP) = λ+(*δ* ^15^N_consumer_−*δ* ^15^N_base_)/2.54 [12], [20], where λ is the trophic position of the organism used as *δ* ^15^N_base_ (in this case λ = 2 for mayflies, considered to be primary consumers), *δ* ^15^N_consumer_ is measured directly, and 2.54 is the average enrichment in *δ* ^15^N per trophic level, appropriate for Australian [42] and tropical [43], [44] consumers, and resembling the average enrichment we observed between our primary and secondary consumers (2.52‰ *δ* ^15^N). We used mayflies from the families Baetidae and Leptophlebiidae as our *δ* ^15^N baseline (with a separate baseline per site) because they are longer-lived than the periphyton that is their main food source [34] and which supports most of the fish biomass in our northern Australian rivers, particularly large-bodied fish [6], [45]. There is evidence that some fish obtain their energy from floodplain periphyton during the wet season to subsidise their river periphyton sources during the dry season, however, these fish still obtain approximately a third of their biomass from river periphyton sources [6]. Further, *δ* ^13^C values of our fish ranged from −36‰ to −13‰, corresponding to the range observed for mayflies across our sites (−41‰ to 14‰), and we found no significant differences in mayfly *δ* ^15^N between the wet and the dry seasons (t_9_ = −1.99, *P* = 0.08), nor between early and late dry seasons (t_16_ = 0.45, *P* = 0.66) at subsets of our sites, indicating there was little variation in our choice of baseline across the spatial and temporal range of our consumers that may have confounded calculations of FCL.

### Measurement of environmental determinants

We used dissolved nutrient concentrations as a proxy for resource availability or productivity (e.g. [20]), which we combined into a single index of productivity. While gross primary productivity would have been a more accurate measure, the large scale of the survey, the distance between sites and the remoteness of terrain meant that only a limited time was able to spent at each location, precluding our ability to obtain such data [34]. Many northern Australian rivers are oligotrophic [46] and their primary production has been shown to be nutrient- rather than light- or carbon-limited [47]–[52]. In the Australian wet-dry tropics and adjacent dryland ecosystems, nutrient limitation of benthic algal production can consequently limit fish production [45], [53]. Algal species are likely to be limited by either N or P, therefore, both N and P are likely to limit production in algal communities and should be considered together [54]. Following Death and Winterbourn [55] and McHugh et al. [27], Principal Components Analysis (PCA) was used to combine measures of total dissolved nitrogen (TDN; <detection limits to 0.1 mg/L) and total dissolved phosphorus (TDP; <detection limits to 0.064 mg/L), obtained from filtered water samples collected from sites at the time of sampling. Both TDN and TDP loaded strongly onto PC1 (both *r*>0.7), which had an eigenvalue over 1.0, and explained 52% of the variation between sites. We retained PC1 as the multivariate index for productivity, adding 10 to each score to ensure they were all positive and that higher values indicated greater productivity [27], [54].

Disturbance was characterised on the basis of hydrological isolation, or period of hydrological disconnection, where longer periods of isolation during the dry season represented higher disturbance [26], [56]. We defined three disturbance levels: low disturbance was represented by perennial sites (i.e. no disconnection), moderate disturbance was represented by intermittent sites that were still flowing at the time of sampling (mid-dry season), and high disturbance was represented by intermittent sites that had already ceased flowing at the time of sampling, and so were disconnected for the longest period. Northern Australia's strongly seasonal wet-dry climate means that the lack of rainfall during the dry season results in most rivers being intermittent and becoming disconnected during the dry season [32]. Both biotic and abiotic conditions in these disconnected waterholes tend to deteriorate with increasing period of hydrological isolation [35], [36]. Therefore, while peak-flow events vary annually in their magnitude and duration and contribute to hydrological variability in these systems [32], [34], [37], we focussed instead on the low-flow events and used the period of hydrological isolation as our measure of disturbance. Because many rivers across northern Australia are ungauged or have limited flow data [32] (only 17 of our 66 sites were gauged), we were unable to use hydrological time series to quantify the period of hydrological isolation at all of our sites. Catchment characteristic such as topography, drainage density and vegetation cover and type can be successfully used as a proxy to classify flow regimes [40], which we matched with the existing ecohydrological classification of gauged rivers in northern Australia [32], and supplemented with local Aboriginal knowledge. We also took into account the flow conditions at the time of sampling, as sites that were already disconnected at the time of sampling during the mid-dry season (May-August) were already disconnected for a longer period than those still flowing (because sites predictably start flowing again in the early wet season, around November). Accordingly, sites were designated along a gradient of increasing disturbance as perennial (n = 25, flowing all the time and representing low disturbance), intermittent flowing (n = 23, intermittent but flowing at the time of sampling, representing moderate disturbance), or intermittent not-flowing (n = 18, these sites had stopped flowing so were hydrologically isolated for the longest period and represented sites of high disturbance). Given we have only defined three levels in our disturbance variable, we also provide a supplementary analysis of the relationship between FCL and the number of zero-flow days, obtained from 20-yr hydrological records from the 17 gauged sites within our total 66 sites.

Ecosystem size, like productivity, was represented by a multivariate index that combined catchment area (0 to 62,000 km^2^), distance from the estuary via watercourse (1 to 695 km), elevation (7 to 521 m.a.s.l.), and active channel width estimated at the time of sampling (<10–1,000 m). This enabled us to include all our sites, including waterholes that received flow inputs as local runoff, sheet flow, or as a variable proportion of overbank flooding from main and distributary channels [35] so their catchment area could not be accurately measured. Principal Components Analysis on the normalised variables resulted in PC1 having the only eigenvalue over 1.0, explaining 62% of the between-site variation, with all variables loading onto it (all *R*>0.6). As we did for the productivity index, we retained the PC1 scores as the multivariate index of ecosystems size, adding 10 to each score to ensure they were all positive [27], [54] and that higher values indicated larger ecosystems, having wider channels and larger catchment areas, together with lower elevations and being closer to the estuary (e.g. main channel sites near river mouth). We also provide a supplementary analysis of the relationship between FCL and catchment area alone, resulting in the exclusion of floodplain waterhole sites (because we could not calculate catchment area for these sites), but providing a relationship allowing direct comparison of the influence of ecosystem size with other published studies (e.g. [26]).

To assess the role of food-web architecture and whether the degree of omnivory would mediate effects of the above mechanisms on FCL, we classified the trophic role of the top predator from each food web (Table S1). At 64 sites, the top predator was one of 23 fish species, and at the remaining four sites it was an invertebrate species. Following Jepsen and Winemiller [57], the trophic class of each fish species was determined from habit, morphology and published summaries of gut contents data [58], [59], and invertebrate consumers were designated a trophic class similarly based on habit, morphology and observational data [60] (M.M. Douglas, *unpublished data*). Nine trophic classes were defined and numerically ranked according to increasing trophic level (Table 2).

**Table 2:** Trophic roles of the top predator in each food web from our 66 sampled sites, along with example species in each group, classified according to increasing trophic level.

Class	Trophic role	Example taxon	Major dietary items					
			Algae & aquatic plants	Detritus	Micro-crustaceans	Macro-invertebrates	Crustaceans	Fish
1	Filtering macroinvertebrates	Philopotamidae		>67%				
2	Predatory macroinvertebrates	Nepidae, Coenagrionidae				>67%		
3	Herbivorous fishes	*Scortum ogilby* (Gulf grunter)	>67%					
4	Benthivorous fishes	*Neosilurus hyrtlii* (Hyrtls tandan)		>33%		>33%		
5	Omnivorous fishes	*Hephaestus fuliginosus* (sooty grunter)	>25%			>33%	>33%	
6	Invertivorous fishes	*Craterocephalus stramineus* (strawman)			>33%	>33%		
7	Insectivorous fishes	*Glossogobius giurus* (flathead goby)				>67%		
8	Generalist predator fishes	*Leiopotherapon unicolor* (spangled perch)				∼30%	∼30%	∼30%
9	Piscivorous fishes	*Strongylura krefftii* (longtom)						>67%

Taxon (macroinvertebrate (n = 4) or fish (n = 23)), habit, morphology, and observational data contributed to defining trophic classes, but designation was largely based on published summaries of gut contents [58], [59] according to relative proportions of major dietary items.

### Data analysis

Relationships among productivity, ecosystem size and trophic role were explored using ordinary least squares (OLS) linear regression. Relationships between these determinants and disturbance (being a categorical variable) were explored using non-parametric analysis of variance and permutation tests of significance on Euclidean distance matrices [61] in PERMANOVA+, the software addition to PRIMER 6 (Primer-E, Plymouth, UK). The data were normally distributed and did not require transformation, nor did they display any spatial autocorrelation. We applied a false discovery rate (FDR) correction to control for the possibility of increased Type I errors associated with multiple tests [62].

We assessed the relative support for each of the determinants (productivity, disturbance, ecosystem size and trophic role of the top predator) hypothesised to control FCL using an information-theoretic model-selection approach [63]. Distance-based linear modelling [61] was performed (using PERMANOVA+), which accommodated using correlated predictor variables and both continuous and categorical variables. Each environmental determinant (normalised) was regressed against the FCL resemblance matrix (Euclidean dissimilarity). The model with the strongest support was identified using values derived from Akaike's Information Criterion corrected for small sample size (AIC_c_), specifically Δ*_i_*, (i.e. = AIC_c*i*_−min[AIC_c_]), Akaike weights *w_i_* (i.e. *w_i_* = *e* ^(−0.5Δ*i*)^/Σ*e* ^(−0.5Δ*i*)^), and the evidence ratio (i.e. *w_top_/w_i_*). Using distance-based linear modelling in PERMANOVA+ also provided a permutation test of significance of the proportion of variation explained by each model.

## Results

At each site we sampled an average of 22±6.6 (SD) consumers and 8.6±2.8 sources. Consumers were represented by 48 fish species and 32 macroinvertebrate taxa. Consumer *δ* ^13^C averaged −27.3±5.0‰ and consumer *δ* ^15^N averaged 6.7±2.7‰ (ranges are presented in Table S1). Food chain length averaged 4.5±0.6, ranging across three trophic levels from 3.2 in a floodplain waterhole to 6.1 in a tributary of the Mitchell River.

Twenty-seven different species represented the top predator across the 66 food webs, and no species was the top predator in more than 12 food webs (Table S1). The generalist predator *Leiopotherapon unicolor* (spangled perch) was the top predator in 12 food webs, the piscivore *Strongylura krefftii* (longtom) was the top predator in 9 food webs, and no other species was the top predator in more than 5 food webs. The most common trophic class of top predator was generalist predators (n = 22 sites), such as *L. unicolor* and *Glossamia aprion* (mouth almighty) that consumed equal proportions of fishes, crustaceans and macroinvertebrates (Table 2). Omnivores (n = 20 sites) were the next most common top predator and included *Hephaestus fuliginosus* (sooty grunter) and *Melanotaenia australis* (rainbowfish) and consumed at least 25% plant material along with crustaceans and macroinvertebrates (Table 2). Piscivores such as *S. krefftii* and *Lates calcarifer* (barramundi) that had a diet dominated by fishes (>67%, Table 2) represented the next most common top predator at 12 sites. Top predators at the remaining 12 sites spanned the remaining six trophic classes (Table S1).

### Relationships among environmental determinants

**Figure 2:**
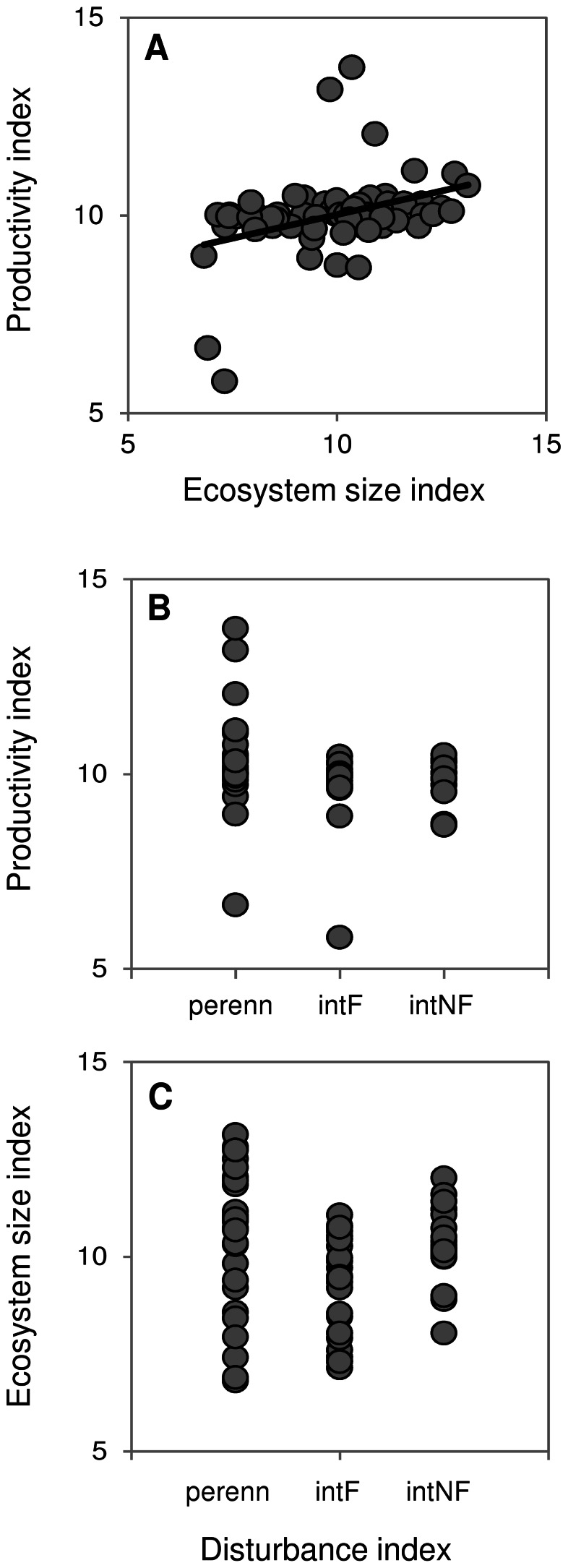
Relationships among environmental determinants. Relationships between A) productivity and ecosystem size (*R^2^* = 0.135, *P*<0.003), B) productivity and disturbance (*R^2^* = 0.070, F_2,63_ = 2.455, *P* = 0.088), and C) ecosystem size and disturbance (*R^2^* = 0.378, F_2,63_ = 4.706, *P*<0.014). For all relationships n = 66 sites. For the disturbance index, “perenn” indicates perennially-flowing sites, “intF” are sites that are intermittent but flowing at the time of sampling, and “intNF” are intermittent non-flowing sites.

The hypothesised determinants of food web structure were not independent of each other in our study, but not in the manner observed in previous studies (e.g. [26], [27]). There was a significantly positive, albeit weak, relationship between productivity (dependent variable) and ecosystem size across our 66 sites (*R^2^* = 0.135, *P* = 0.003), where larger ecosystems were more productive (Fig. 2A), but there was no significant relationship between productivity and disturbance (*R^2^* = 0.070, F_2,63_ = 2.455, *P* = 0.088; Fig. 2B). We found a significant U-shaped relationship between ecosystem size (dependent variable) and disturbance (*R^2^* = 0.378, F_2,63_ = 4.706, *P*<0.014), where ecosystems experiencing low disturbance or high disturbance were larger than those experiencing moderate disturbance (Fig. 2C). Trophic class was not predictable from productivity (*R^2^* = 0.052, *P* = 0.080), ecosystem size (*R^2^* = 0.029, *P* = 0.170), or disturbance (*R^2^* = 0.268, F_2,63_ = 2.542, *P* = 0.089).

### Relationships between environmental determinants and food chain length

**Figure 3:**
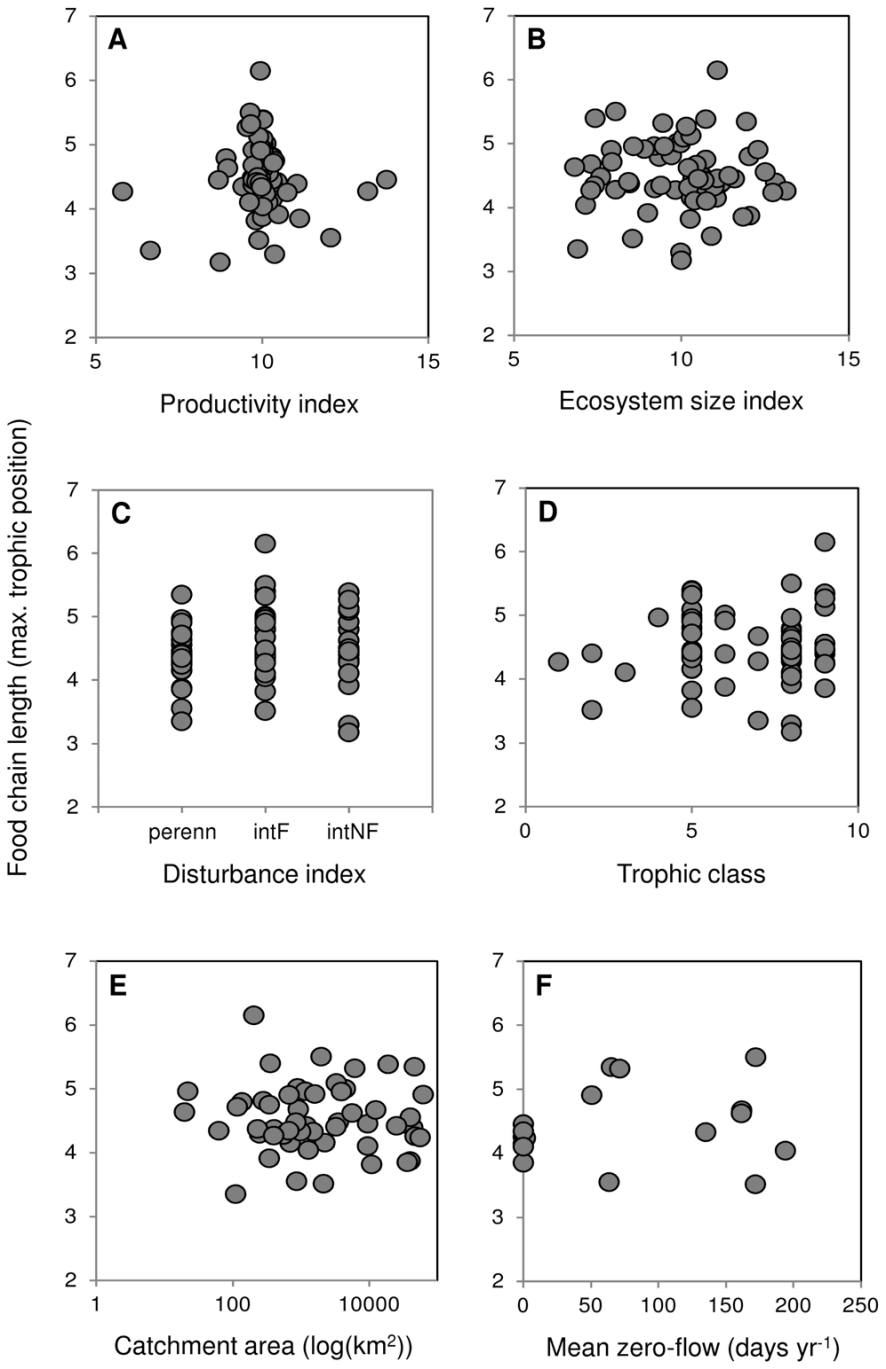
Relationships between environmental determinants and food chain length. Relationships between FCL and A) productivity (*R^2^* = 0.000, *P* = 0.914), B) ecosystem size (*R^2^* = 0.000, *P* = 0.927), C) disturbance (*R^2^* = 0.019, F_2,63_ = 2.429, *P* = 0.098), and D) trophic class of the top predator (*R^2^* = 0.003, *P* = 0.681). These relationships all had n = 66, and the disturbance categories are labelled as in Fig. 2. Also presented are supplementary relationships between FCL and E) catchment area (n = 54, *R^2^* = 0.001, *P* = 0.785), and F) the mean annual number of zero-flow days (n = 17, *R^2^* = 0.023, *P* = 0.561).

Variation in FCL was best explained by disturbance, which had the lowest AIC_c_ and represented 65% of model weight (Table 3). However, it only explained 7% of variation in FCL among sites: none of the environmental determinants, including disturbance, explained a significant proportion of variation in FCL (Table 3). This was reflected by the lack of a relationship between FCL and productivity (*R^2^* = 0.000, *P* = 0.914; Fig. 3A), ecosystem size (*R^2^* = 0.000, *P* = 0.927; Fig. 3B), disturbance (*R^2^* = 0.019, F_2,63_ = 2.429, *P* = 0.098; Fig. 3C) and the trophic class of the top predator (*R^2^* = 0.003, *P* = 0.681; Fig. 3D). Our supplementary analyses also showed no significant relationship between catchment area and FCL (n = 54, *R^2^* = 0.001, *P* = 0.785; Fig. 3E), and no significant relationship between the number of zero-flow days and FCL (n = 17, *R^2^* = 0.023, *P* = 0.561; Fig. 3F).

**Table 3:** Model selection results for evaluating the hypothesised determinants of food chain length.

Hypothesised determinant	AIC_c_	Δ*_i_*	*w_i_*	Evidence ratio	*R* ^2^	p
Productivity	−76.32	2.69	0.17	3.82	<0.01	0.913
Disturbance	−79.01	0.00	0.65	1.00	0.07	0.105
Ecosystem size	−76.31	2.70	0.17	3.85	<0.01	0.925
Trophic role of top predator	−69.50	9.51	0.01	166.67	0.14	0.326

AIC_c_ is Akaike's Information Criterion corrected for small sample size, Δ*_i_* is the AIC_c_ difference between a given model and that with the lowest AIC_c_ value, and *w_i_* is the Akaike weight. The evidence ratio is the relative weight compared to the top model. *R* ^2^ is the coefficient of determination, and the p-value is the significance of the proportion of variation explained by each determinant as assessed by marginal permutation tests.

## Discussion

Contrary to our expectations and the well-established patterns in the literature [25] (Table 1), we found none of the classic environmental determinants had any relationship with FCL, nor was FCL related to the trophic role of the top predator. This was despite our large sample size and the considerable variation in FCL among our food webs. Food chain length in our food webs averaged 4.5±0.6, which does not support previous predictions of short food chains in these wet-dry tropical rivers [31], but is still within the range observed in aquatic systems elsewhere [44], [64]. The effects of environmental determinants tend to appear at a local scale (e.g. [64]) and we surmise that larger, regional-scale processes might be driving our observed variation in FCL, as theoretically demonstrated by previous authors [23], [65]. We hypothesise that the hydrological reconnection via seasonal inundation across large tracts of the landscape, even if brief (e.g. [6]), effectively “opens” these food webs and buffers the effects of local environmental determinants on FCL.

Productivity was not related to FCL, suggesting that the availability of resources is not an important driver of food web structure in these wet-dry tropical ecosystems. Other studies that found no effect of productivity simultaneously found a positive effect of ecosystem size on FCL (Table 1), suggesting that spatial considerations are a more important influence on food web structure [20], [26], [29]. Our results did not support this suggestion. It is possible that our use of nutrient concentrations was too insensitive a measure of productivity, as they indicate total production rather than the proportion of production that is actually available to consumers [66]. However, we also found that FCL was also unrelated to benthic chlorophyll *a* at a subset of sites (n = 26, *R^2^* = 0.01, *P* = 0.515; T.D. Jardine, *unpublished data*). Benthic algae are known to be an important, if not dominant, energy source supporting fish biomass in Australia's northern and dryland rivers [31], [45], [53], and their biomass is positively related to gross primary productivity [53] (N.E. Pettit, *unpublished data*). Furthermore, nutrient availability limits primary producer biomass in these rivers, more so than light or carbon [47], [51], [52], so we considered nutrient concentrations to be an appropriate proxy for productivity, consistent with other studies on FCL (e.g. [20]). However, we also note that our nutrient concentrations were low, typical of northern Australian rivers [46], so either we did not have a large enough gradient to show a relationship with FCL (despite the considerable variation in FCL), or these low concentrations indicate rapid nutrient turnover and sufficient nutrient supply [49], such that there is no limitation of FCL.

We found no influence of disturbance, as measured by the degree of hydrological isolation, on FCL. Longer food chains are hypothesised to be less resilient to disturbance than short food chains [17], hence systems experiencing larger or more frequent disturbances are predicted to have shorter food chains. Research that has tested the influence of disturbance on FCL has found either neutral or negative effects (Table 1), and on average, no effect [25]. In the only study to have experimentally manipulated disturbance, Walters and Post [67] found no effect of low-flow disturbance on FCL in stream food webs; the authors suggested that local refugia mitigate disturbance effects, a conclusion also reached by Townsend et al. [68]. This is entirely possible in Australia's northern rivers, where isolated waterholes, both in-channel and floodplain, can represent the only aquatic habitat in the landscape and are thus a critical refuge during the dry season for a range of biota [37], [69]. However, both abiotic and biotic conditions in these waterholes tend to deteriorate over the course of the dry season as available habitat contracts, such that their “refuge quality” is markedly reduced [35], [36], our rationale for considering hydrological isolation a disturbance.

One of the mechanisms proposed for the disturbance hypothesis is that disturbance results in the loss of top predators, shortening food chains [18], [19], [26]. In a related analysis of biotic assemblage structure across our sites, we found that fish diversity was lower at intermittent than at perennial sites [33], potentially supporting this mechanism. However, we found no relationship between disturbance and the trophic role of the top predator, indicating that although species may be lost from the food web in more hydrologically isolated sites, they are not necessarily top piscivorous predators, and the trophic levels represented by the remaining species are equivalent to those represented at less disturbed, perennial sites. Research on macroinvertebrate assemblages from other wet-dry rivers in northern Australia indicates that although biodiversity is influenced by the degree of intermittency, generalist feeding strategies result in food web structure being buffered from hydrological disturbance [70]. The modelling that led to the hypothesis that longer food chains are less resilient [17] was based on the assumption that only basal species show self-regulation, i.e. intraspecific interactions that negatively affect population size. By extending the assumption of self-regulation to higher trophic levels, a more realistic assumption accommodating density-dependent feedback and intraspecific competition etc., Sterner et al. [71] showed that longer food chains are actually *more* resilient. This suggests that longer food chains are not less stable and, theoretically, not limited by disturbance, potentially explaining the lack of a clear effect on FCL in the broader literature [11], [25] and supported by our findings here.

We also found no effect of ecosystem size on FCL, an unexpected result given that most studies which have tested ecosystem size have found a positive effect on FCL [25]. The ecosystem size hypothesis has more support in the literature than either the productivity or disturbance hypotheses (Table 1), although variability in effect magnitude has led to predictions that field tests of ecosystem size may find non-positive effects on FCL [25], a prediction our findings confirm. As outlined earlier, there are numerous mechanisms proposed to explain the influence of ecosystem size. The productive space hypothesis predicts larger ecosystems have more resources and therefore support longer food chains [22]. Although we had a positive relationship between productivity and ecosystem size, FCL did not show a positive relationship with either determinant, so our findings do not support this mechanism. Larger ecosystems are hypothesised to support greater functional trophic diversity and less omnivory [20], a mechanism also not supported by our findings as there was no relationship between ecosystem size and the degree of omnivory shown by the top predator.

Larger ecosystems can support more species, suggested to result in longer food chains [21]. Assemblage composition data collected during this research indicated that fish assemblages (but not macroinvertebrate or vegetation assemblages) in northern Australia can be more species-rich at perennial than at intermittent sites [33], and perennial sites were more likely to be larger on average (Fig. 2C). However, there was more variability in ecosystem size among perennial sites, and non-flowing intermittent sites also represented larger ecosystems (Fig. 2C) but did not show related increases in species richness [33]. Further, there was no relationship between FCL and the number of consumers in each food web (n = 66, *R^2^* = 0.013, *P* = 0.369), indicating species richness, via ecosystem size, did not contribute to our observed variation in FCL.

Related to species richness are the mechanisms of enhanced colonisation opportunity and the promotion of predator-prey co-existence that may explain the influence of ecosystem size on FCL [15], [23]. These mechanisms suggest that larger ecosystems are better able to support intraguild predation and longer food chains so long as the intraguild prey are not limited in their dispersal and are good colonisers [15]. Our complementary analysis of community assembly at a subset of the study sites (n = 46) has found that dispersal limitation is not a strong factor structuring biotic assemblages [33], supporting this prediction, but the absence of a relationship between ecosystem size and FCL here does not support this mechanism of the ecosystem size hypothesis.

Underpinning many of the above hypotheses explaining effects on FCL are references to food web architecture, i.e. proximate structural mechanisms. These mechanisms suggest that the degree of dietary specialisation (e.g. piscivory) versus omnivory in the top predator is likely to modify FCL itself, or mediate the effects of environmental determinants on FCL [15], [28]. We found that generalist predators and omnivores were equally among the most common trophic roles displayed by top predators in our food webs (n = 22 and 20, respectively), but this did not alter observed FCL, nor did it modify the effects of any environmental determinant on FCL. Previous research suggests that ecosystem size increases FCL because larger-bodied top predators tend to be absent from smaller ecosystems [20], [27], [28], or that the insertion of new species at lower trophic levels increases the trophic position of the top predator [28], [29]. These mechanisms depend on food webs having a strong size-structure, where top predators are notably larger than prey from lower trophic levels [44]. The food webs in our wet-dry tropical rivers are not strongly size-structured: for example, piscivorous longtom (*Strongylura krefftii*) are long, slender with elongated jaws and generally reach 500 mm SL whereas the widespread generalist predator spangled perch (*Leiopotherapon unicolor*) is a robust species often only reaching 150 mm SL [58]. Omnivorous fish also show a range of sizes and include sooty grunter (*Hephaestus fuliginosus*), a moderately deep-bodied fish commonly up to 350 mm SL, and rainbowfish (*Melanotaenia* spp.) that grow to about 100 mm SL. A common prey fish is the largely herbivorous bony bream or gizzard shad (*Nematolosa erebi*), a deep-bodied fish commonly 150–300 mm SL [58]. The weak size-structure of our food webs mirrors that observed in food webs from the Neotropics [43], [44] and suggests that if larger-bodied fish are absent from smaller ecosystems, these fish are not necessarily predators and could be from a number of trophic levels, and so are unlikely to show relationships with FCL [43]. Another feature of such reticulate but weakly size-structured food webs is widespread omnivory [72], where predators can consume from multiple trophic levels so increases in FCL are likely to occur through the insertion of intermediate trophic levels rather than the addition of new top predators [15], [44]. This may be what is occurring in our food webs, where those with longer food chains have more intermediate trophic levels, however, as noted above, we found no relationship with FCL and the number of consumers.

It is possible that the fish we sampled are actually not apical predators in our food webs. Large-bodied predators such as elasmobranchs, crocodiles and piscivorous waterbirds can also be present in this landscape, potentially increasing FCL. While quantitative sampling of these consumers was beyond the scope of the present study, opportunistic sampling of freshwater crocodiles (*Crocodylus johnstoni*) from four sites and bull sharks (*Carcharhinus leucas*) from two sites indicated these predators occupied an equivalent trophic position to piscivorous fishes. But waterbirds, sampled opportunistically from 23 sites, often had more enriched *δ* ^15^N than piscivorous fishes (up to 5‰ more enriched, D.M. Warfe, *unpublished data*). Piscivorous waterbirds may therefore occupy a trophic level higher than piscivorous fish, but are not restricted to aquatic habitats so have the capacity to link food webs across larger spatial scales than fish, a possibility which supports the proposed scale-invariance of food web architecture [2]. However, food webs that include waterbirds are effectively open and less likely still to respond to local environmental determinants.

We conclude that our inability to identify environmental factors explaining the observed variation in FCL among our food webs is due to regional processes [23], [65] and a degree of plasticity in trophic dynamics. Both fish and invertebrate consumers from northern Australia can show considerable variation in diet, potentially allowing them to take advantage of scarce resources during the dry season when aquatic habitats are greatly contracted [31], [58], [59], [70], as well as abundant resources during the wet season [6]. While limited dispersal has been theoretically shown to limit FCL at a metacommunity scale [65], associated research in this landscape has shown that dispersal limitation plays only a minor role in species assembly [33] and that floodplain carbon contributes to the biomass of predatory fish caught in permanent waterbodies [6], suggesting that fishes are not restricted in their capacity to move across the landscape.

The seasonal hydrological connection of rivers and floodplains across the landscape, even if relatively brief, can facilitate the movement of fishes onto the floodplain during the wet season where they feed and grow, thereby subsidising stream and river food webs during the dry season and temporarily linking spatially disparate food webs [2], [6], [73]. We propose that such seasonal linkage creates a “meta-foodweb” during the wet season, which, like a metacommunity [74], could be considered as a set of local food webs that are connected by the landscape-scale movements of high-order consumers. This meta-foodweb then splits into sub-foodwebs as sites become hydrologically disconnected during the dry season, preventing the movement of consumers. This can lead to stochasticity in assemblage structure among sites, similar to that observed in Neotropical river-floodplain systems [75], such that the number (and type) of trophic groups represented is variable, leading to variability in FCL. This hypothesis supports theoretical predictions that mobile consumers that are able to respond to, and exploit, spatial variability in resources can counteract the destabilising effects of local perturbations and thereby confer stability and persistence to food-web dynamics [2], [7], [8], [76], [77]. We suggest that the seasonal hydrological reconnection is a predominant influence on food web structure in these wet-dry tropical systems [31], [37], overriding local effects of productivity, disturbance and ecosystem size, and potentially conferring resilience to the structure of biotic assemblages and food webs [36].

Wet-dry tropical regions cover extensive areas across South America, Africa, India and southeast Asia, representing a large fraction of the earth's land area, so the occurrence of meta-foodwebs linked by seasonal hydrological connectivity and fish movement could potentially be relatively widespread [73]. The corollary to this is that structures (e.g., dams and levees) and processes (e.g., flow regime alteration and saltwater intrusion) that disrupt the timing, duration and frequency of hydrological connectivity across the landscape, and thereby reduce the capacity of fish to reconnect food webs, may lead to food web structure becoming less resilient to anthropogenic perturbations.

## Supporting Information

Figure S1 **Photos of selected sampling sites.**
(DOC)

Table S1 **Ranges (and consumer identity) of** ***δ*** **^13^C and** ***δ*** **^15^N values, and the trophic class of top consumers, from each food web.**
(DOC)
