# Supplementary material for: Productivity, Disturbance and Ecosystem Size Have No Influence on Food Chain Length in Seasonally Connected Rivers
Source: PLoS One. 2013 Jun 12;8(6):e66240. doi: 10.1371/journal.pone.0066240 (PMC3680379; doi:10.1371/journal.pone.0066240)
Supplement: Figure S1 — Photos of selected sampling sites. (DOC) [file pone.0066240.s001.doc]

**PLOS One – Supporting Information**

**Figure S1** Photos of selected sampling sites

D.M. Warfe, T.D. Jardine, N.E. Pettit, S.K. Hamilton, B.J. Pusey, S.E. Bunn, P.M. Davies & M.M. Douglas. Productivity, disturbance and ecosystem size have no effect on food chain length in seasonally connected rivers.

**Figure S1.** **Photos of selected sampling sites.** These photos provide examples of perennially connected sites (n = 25; left column), intermittent sites that were flowing (n = 23; middle column), and intermittent sites that were not flowing (n = 18; right column) at the time of sampling (May-August 2008). A) Daly River main channel, B) Fitzroy River upper main channel, C & H) Fitzroy River tributaries (Margaret River and Mt Pierre Creek, respectively), D, E & G) Daly River tributaries (Hayes Creek, Fergusson and Katherine Rivers, respectively), F) Daly River floodplain waterhole (Milkwood Lagoon), and I) Fitzroy River floodplain waterhole (Sandy Billabong). Photo credits: D.M. Warfe.
