# Supplementary material for: Productivity, Disturbance and Ecosystem Size Have No Influence on Food Chain Length in Seasonally Connected Rivers
Source: PLoS One. 2013 Jun 12;8(6):e66240. doi: 10.1371/journal.pone.0066240 (PMC3680379; doi:10.1371/journal.pone.0066240)
Supplement: Table S1 — Ranges (and consumer identity) of δ 13C and δ 15N values, and the trophic class of top consumers, from each food web. (DOC) [file pone.0066240.s002.doc]

**PLOS One – Supporting Information**

**Table S1** Ranges (and consumer identity) of *δ*^13^C and *δ*^15^N values, and the trophic class of top consumers, from each food web.

D.M. Warfe, T.D. Jardine, N.E. Pettit, S.K. Hamilton, B.J. Pusey, S.E. Bunn, P.M. Davies & M.M. Douglas. Productivity, disturbance and ecosystem size have no effect on food chain length in seasonally connected rivers.

**Table S1.** Ranges (and consumer identity) of *δ*^13^C and *δ*^15^N values in each food web, and the trophic class of top consumers (identified by maximum trophic position), from each site in northern Australia.

| ***δ*^13^C** |  |  |  |  |
| --- | --- | --- | --- | --- |
| **Site** | **Min.** | **Taxon** | **Max.** | **Taxon** |
| **Daly River catchment** |  |  |  |  |
| Bradshaw Creek | -40.8 | Baetidae (Ephemeroptera) | -21.1 | *Leiopotherapon unicolor* (spangled perch) |
| Brandy Bottle Creek | -30.1 | Zygoptera (Odonata) | -22.4 | *Leiopotherapon unicolor* (spangled perch) |
| Chilling Creek | -37.4 | Leptophlebiidae (Ephemeroptera) | -20.5 | *Leiopotherapon unicolor* (spangled perch) |
| Daly River (Beeboom) | -39.0 | Corbiculidae (Gastropoda) | -12.8 | *Himantura chaophraya* (whipray) |
| Daly River (Claravale) | -38.5 | Philopotamidae (Trichoptera) | -20.1 | *Lates calcarifer* (barramundi) |
| Daly River (Mt Nancar) | -33.7 | Zygoptera (Odonata) | -17.3 | *Carcharhinus leucas* (bull shark) |
| Daly River (Oolloo) | -40.5 | Pyralidae (Lepidoptera) | -22.9 | *Arius graeffei* (fork-tailed catfish) |
| Daly River (upstream Oolloo) | -40.2 | Baetidae (Ephemeroptera) | -22.0 | *Macrobrachium spinipes* (Decapoda) |
| Douglas River Hot Springs | -34.3 | Pyralidae (Lepidoptera) | -22.4 | *Leiopotherapon unicolor* (spangled perch) |
| Edith River | -32.4 | Philopotamidae (Trichoptera) | -23.1 | *Macrobrachium spinipes* (Decapoda) |
| Fergusson River | -31.9 | Leptophlebiidae (Ephemeroptera) | -21.2 | *Leiopotherapon unicolor* (spangled perch) |
| Flora River | -32.2 | Philopotamidae (Trichoptera) | -19.7 | *Leiopotherapon unicolor* (spangled perch) |
| Grace Creek | -33.7 | Baetidae (Ephemeroptera) | -19.5 | Nepidae (Hemiptera) |
| Green Ant Creek | -37.5 | Baetidae (Ephemeroptera) | -22.5 | *Amniataba percoides* (barred grunter) |
| Hayes Creek | -38.6 | Baetidae (Ephemeroptera) | -23.8 | *Melanotaenia australis* (rainbowfish) |
| Katherine River (Galloping Jacks) | -36.8 | Baetidae (Ephemeroptera) | -22.8 | *Leiopotherapon unicolor* (spangled perch) |
| Katherine River (Manyallalak) | -34.8 | Philopotamidae (Trichoptera) | -20.7 | *Hephaestus fuliginosus* (sooty grunter) |
| King River | -33.1 | Hydropsychidae (Trichoptera) | -19.7 | *Leiopotherapon unicolor* (spangled perch) |
| Kowai Lagoon | -31.9 | Leptoceridae (Trichoptera) | -22.0 | *Oxyeleotris selheimi* (giant cod) |
| Limestone Creek | -40.5 | Baetidae (Ephemeroptera) | -20.5 | *Leiopotherapon unicolor* (spangled perch) |
| Mango Farm Billabong | -39.0 | Leptophlebiidae (Ephemeroptera) | -22.1 | Belostomatidae (Hemiptera) |
| Mathison Creek | -37.5 | Hydropsychidae (Trichoptera) | -21.0 | *Leiopotherapon unicolor* (spangled perch) |
| Middle Creek | -41.6 | Pyralidae (Lepidoptera) | -21.8 | *Glossamia aprion* (mouth almighty) |
| Mission Hole Lagoon | -33.2 | Hyriidae (Gastropoda) | -23.8 | Lymnaeidae (Gastropoda) |
| Red Lily Lagoon | -29.0 | *Macrobrachium spinipes* (Decapoda) | -21.3 | *Mogurnda mogurnda* (gudgeon) |
| Stray Creek | -34.5 | Baetidae (Ephemeroptera) | -21.0 | Dytiscidae adult (Coleoptera) |
| **Mitchell River catchment** |  |  |  |  |
| Bushy Creek | -30.1 | Leptophlebiidae (Ephemeroptera) | -19.7 | *Leiopotherapon unicolor* (spangled perch) |
| Cairo Lagoon | -33.8 | zooplankton | -17.8 | Gastropoda |
| Dickson Hole | -32.1 | Baetidae (Ephemeroptera) | -25.1 | *Leiopotherapon unicolor* (spangled perch) |
| Emu Creek | -33.3 | Gomphidae (Odonata) | -20.7 | *Strongylura krefftii* (longtom) |
| Fishhole Creek | -38.8 | Culicidae (Diptera) | -21.0 | *Scleropages jardinii* (saratoga) |
| Hodgkinson River | -38.4 | Hyriidae (Gastropoda) | -20.1 | *Leiopotherapon unicolor* (spangled perch) |
| Kingfish Lagoon | -28.5 | zooplankton | -15.0 | Baetidae (Ephemeroptera) |
| Lynd River (Lyndbrook) | -33.8 | Caenidae (Ephemeroptera) | -23.2 | *Leiopotherapon unicolor* (spangled perch) |
| Magnificent Creek | -40.0 | Caenidae (Ephemeroptera) | -26.5 | *Leiopotherapon unicolor* (spangled perch) |
| McLeod River | -35.8 | Coenagrionidae (Odonata) | -24.6 | *Leiopotherapon unicolor* (spangled perch) |
| Mitchell River (Gamboola) | -36.7 | Hyriidae (Gastropoda) | -22.3 | *Lates calcarifer* (barramundi) |
| Mitchell River (Koolatah) | -35.4 | Hyriidae (Gastropoda) | -20.6 | *Strongylura krefftii* (longtom) |
| Mitchell River (Cooktown) | -34.4 | Baetidae (Ephemeroptera) | -21.7 | Pleidae (Hemiptera) |
| Palmer River (Drumduff) | -40.9 | Diptera | -21.2 | *Arius graeffei* (fork-tailed catfish) |
| Palmer River (Goldfields) | -31.3 | Leptophlebiidae (Ephemeroptera) | -22.2 | *Leiopotherapon unicolor* (spangled perch) |
| Rifle Creek | -40.3 | Baetidae (Ephemeroptera) | -22.2 | *Leiopotherapon unicolor* (spangled perch) |
| Saltwater Creek | -34.0 | Caenidae (Ephemeroptera) | -22.2 | *Leiopotherapon unicolor* (spangled perch) |
| Tate River (Ootan) | -31.9 | Baetidae (Ephemeroptera) | -21.7 | *Leiopotherapon unicolor* (spangled perch) |
| Ten Mile Lagoon | -35.3 | zooplankton | -20.8 | *Oxyeleotris selheimi* (giant cod) |
| Twelve Mile Lagoon | -30.2 | Hydracarina (Arachnida) | -21.2 | *Oxyeleotris lineolatus* (sleepy cod) |
| Walsh River (Nullinga) | -33.1 | Leptophlebiidae (Ephemeroptera) | -11.6 | Corixidae (Hemiptera) |
| Walsh River (Rookwood) | -26.7 | Dytiscidae adult (Coleoptera) | -23.4 | Baetidae (Ephemeroptera) |
| **Fitzroy River catchment** |  |  |  |  |
| Annie Creek | -35.3 | Pyralidae (Lepidoptera) | -23.3 | Nepidae (Hemiptera) |
| Adcock River | -31.6 | Hydropsychidae (Trichoptera) | -24.8 | *Melanotaenia australis* (rainbowfish) |
| Barnett River | -29.0 | Helicopsychidae (Trichoptera) | -20.9 | *Hephaestus jenkinsi* (Jenkins grunter) |
| Bayulu waterhole | -25.7 | zooplankton | -16.7 | Baetidae (Ephemeroptera) |
| Brooking Creek | -29.0 | Leptophlebiidae (Ephemeroptera) | -22.8 | Belostomatidae (Hemiptera) |
| Fitzroy River (Bayulu) | -33.7 | Hyriidae (Gastropoda) | -20.9 | *Neoarius midgleyi* (shovel-nosed catfish) |
| Fitzroy River (Bluebush) | -29.5 | Nepidae (Hemiptera) | -22.5 | *Melanotaenia australis* (rainbowfish) |
| Fitzroy River (Noonkanbah) | -35.5 | Hyriidae (Gastropoda) | -20.3 | *Arius graeffei* (fork-tailed catfish) |
| Fossil Downs waterhole | -25.3 | zooplankton | -16.4 | *Toxotes chatareus* (archerfish) |
| Gillagoowa Spring | -32.9 | Hydropsychidae (Trichoptera) | -21.6 | *Oxyeleotris selheimi* (giant cod) |
| Hann River | -31.6 | Leptoceridae (Trichoptera) | -22.4 | *Melanotaenia australis* (rainbowfish) |
| Manning River | -30.2 | Calamoceratidae (Trichoptera) | -21.5 | *Macrobrachium spinipes* (Decapoda) |
| Margaret River (Yiyili) | -30.9 | *Nematolosa erebi* (bony bream) | -23.8 | *Leiopotherapon unicolor* (spangled perch) |
| Margaret River (Muludja) | -33.1 | Hyriidae (Gastropoda) | -23.6 | *Toxotes chatareus* (archerfish) |
| Mary River | -30.0 | Leptoceridae (Trichoptera) | -21.3 | *Glossogobius giurus* (flathead goby) |
| Mt Pierre Creek (Galeru Gorge) | -35.2 | Hydropsychidae (Trichoptera) | -17.0 | *Leiopotherapon unicolor* (spangled perch) |
| Traine River | -32.2 | Hyriidae (Gastropoda) | -23.5 | *Neosilurus hyrtlii* (Hyrtls tandan) |
| Troys Lagoon | -20.8 | Leptoceridae (Trichoptera) | -15.6 | Gastropoda |

| ***δ*^15^N** |  |  |  |  |  |
| --- | --- | --- | --- | --- | --- |
| **Site** | **Min.** | **Taxon** | **Max.** | **Taxon** | **Trophic class of top predator** |
| **Daly River catchment** |  |  |  |  |  |
| Bradshaw Creek | 2.4 | Calamoceratidae (Trichoptera) | 9.2 | *Oxyeleotris lineolatus* (sleepy cod) | generalist predator |
| Brandy Bottle Creek | 1.6 | Caenidae (Ephemeroptera) | 8.7 | *Oxyeleotris lineolatus* (sleepy cod) | generalist predator |
| Chilling Creek | 2.6 | Leptophlebiidae (Ephemeroptera) | 8.7 | *Leiopotherapon unicolor* (spangled perch) | generalist predator |
| Daly River (Beeboom) | 4.6 | Leptoceridae (Trichoptera) | 11.1 | *Craterocephalus stramineus* (strawman) | invertivore |
| Daly River (Claravale) | 5.8 | Hydrophilidae (Coloeptera) | 10.8 | *Lates calcarifer* (barramundi) | piscivore |
| Daly River (Mt Nancar) | 5.7 | Baetidae (Ephemeroptera) | 11.4 | *Carcharhinus leucas* (bull shark) | piscivore |
| Daly River (Oolloo) | 3.1 | zooplankton | 12.0 | *Strongylura krefftii* (longtom) | piscivore |
| Daly River (upstream Oolloo) | 4.4 | zooplankton | 10.5 | *Craterocephalus stramineus* (strawman) | invertivore |
| Douglas River Hot Springs | 3.3 | Leptophlebiidae (Ephemeroptera) | 8.8 | *Amniataba percoides* (barred grunter) | omnivore |
| Edith River | 1.9 | Nepidae (Hemiptera) | 8.5 | *Glossamia aprion* (mouth almighty) | generalist predator |
| Fergusson River | -3.2 | Ecnomidae (Trichoptera) | 8.5 | *Macrobrachium spinipes* (Decapoda) | omnivore |
| Green Ant Creek | 2.1 | Simuliidae (Diptera) | 10.2 | *Macrobrachium spinipes* (Decapoda) | omnivore |
| Hayes Creek | 2.0 | Caenidae (Ephemeroptera) | 8.7 | *Amniataba percoides* (barred grunter) | omnivore |
| Limestone Creek | 1.8 | Baetidae (Ephemeroptera) | 9.4 | *Macrobrachium spinipes* (Decapoda) | omnivore |
| Grace Creek | 2.0 | Leptophlebiidae (Ephemeroptera) | 10.1 | *Ambassis macleayi* (glassfish) | invertivore |
| Flora River | 2.5 | Leptoceridae (Trichoptera) | 8.1 | *Macrobrachium spinipes* (Decapoda) | omnivore |
| Katherine River (Galloping Jacks) | 4.0 | Tabanidae (Diptera) | 11.2 | *Neoarius midgleyi* (shovel-nosed catfish) | piscivore |
| Katherine River (Manyallalak) | 2.0 | Leptophlebiidae (Ephemeroptera) | 9.9 | *Hephaestus fuliginosus* (sooty grunter) | omnivore |
| King River | 4.6 | Leptoceridae (Trichoptera) | 9.6 | *Macrobrachium spinipes* (Decapoda) | omnivore |
| Kowai Lagoon | -2.1 | Pyralidae (Lepidoptera) | 9.3 | *Leiopotherapon unicolor* (spangled perch) | generalist predator |
| Mango Farm Billabong | 3.9 | Caenidae (Ephemeroptera) | 10.8 | *Strongylura krefftii* (longtom) | piscivore |
| Mathison Creek | 1.1 | Caenidae (Ephemeroptera) | 8.4 | *Leiopotherapon unicolor* (spangled perch) | generalist predator |
| Middle Creek | 2.8 | Hydrophilidae (Coloeptera) | 9.8 | *Leiopotherapon unicolor* (spangled perch) | generalist predator |
| Mission Hole Lagoon | 4.3 | Caenidae (Ephemeroptera) | 10.5 | *Strongylura krefftii* (longtom) | piscivore |
| Red Lily Lagoon | 1.4 | Caenidae (Ephemeroptera) | 8.5 | *Melanotaenia australis* (rainbowfish) | omnivore |
| Stray Creek | 2.9 | Leptophlebiidae (Ephemeroptera) | 9.3 | *Leiopotherapon unicolor* (spangled perch) | generalist predator |
| **Mitchell River catchment** |  |  |  |  |  |
| Bushy Creek | 3.9 | Leptophlebiidae (Ephemeroptera) | 10.6 | *Hephaestus carbo* (coal grunter) | generalist predator |
| Cairo Lagoon | 2.7 | Hydrophilidae (Coleoptera) | 9.6 | *Glossamia aprion* (mouth almighty) | generalist predator |
| Dickson Hole | -1.3 | Leptoceridae (Trichoptera) | 8.0 | *Hephaestus fuliginosus* (sooty grunter) | omnivore |
| Emu Creek | 2.0 | Baetidae (Ephemeroptera) | 8.5 | *Strongylura krefftii* (longtom) | piscivore |
| Fishhole Creek | 2.4 | Culicidae (Diptera) | 9.7 | *Scleropages jardinii* (saratoga) | generalist predator |
| Hodgkinson River | 5.6 | Hydrophilidae (Coleoptera) | 11.3 | Coenagrionidae (Odonata) | predatory macroinvertebrate |
| Kingfish Lagoon | 2.8 | Pleidae (Hemiptera) | 12.3 | *Strongylura krefftii* (longtom) | piscivore |
| Lynd River (Lyndbrook) | 0.0 | Calamoceratidae (Trichoptera) | 7.3 | *Leiopotherapon unicolor* (spangled perch) | generalist predator |
| Magnificent Creek | 1.3 | Baetidae (Ephemeroptera) | 12.3 | *Strongylura krefftii* (longtom) | piscivore |
| McLeod River | 1.0 | Calamoceratidae (Trichoptera) | 8.8 | *Leiopotherapon unicolor* (spangled perch) | generalist predator |
| Mitchell River (Cooktown) | 2.6 | Hydroptilidae (Trichoptera) | 10.9 | Nepidae (Hemiptera) | predatory macroinvertebrate |
| Mitchell River (Gamboola) | 5.7 | Baetidae (Ephemeroptera) | 12.3 | *Hephaestus fuliginosus* (sooty grunter) | omnivore |
| Mitchell River (Koolatah) | 2.7 | Pleidae (Hemiptera) | 11.4 | *Strongylura krefftii* (longtom) | piscivore |
| Palmer River (Drumduff) | 4.0 | Pleidae (Hemiptera) | 10.7 | *Scortum ogilby* (Gulf grunter) | herbivore |
| Palmer River (Goldfields) | 0.1 | Leptoceridae (Trichoptera) | 9.2 | *Pingalla gilberti* (Gilbert’s grunter) | omnivore |
| Rifle Creek | 4.2 | Calamoceratidae (Trichoptera) | 9.2 | *Mogurnda mogurnda* (gudgeon) | insectivore |
| Saltwater Creek | -1.2 | Caenidae (Ephemeroptera) | 6.8 | *Melanotaenia splendida* (rainbowfish) | omnivore |
| Tate River (Ootan) | 1.3 | Baetidae (Ephemeroptera) | 10.3 | *Leiopotherapon unicolor* (spangled perch) | generalist predator |
| Ten Mile Lagoon | 0.3 | Corixidae (Hemiptera) | 9.4 | *Glossamia aprion* (mouth almighty) | generalist predator |
| Twelve Mile Lagoon | -0.3 | Pyralidae (Lepidoptera) | 10.5 | *Strongylura krefftii* (longtom) | piscivore |
| Walsh River (Nullinga) | 4.2 | Dytiscidae adult (Coleoptera) | 13.7 | Philopotamidae (Trichoptera) | filtering macroinvertebrate |
| Walsh River (Rookwood) | 1.0 | Baetidae (Ephemeroptera) | 9.4 | *Macrobrachium spinipes* (Decapoda) | omnivore |
| **Fitzroy River catchment** |  |  |  |  |  |
| Annie Creek | 0.8 | Gastropoda | 7.5 | *Oxyeleotris selheimi* (giant cod) | generalist predator |
| Adcock River | -0.2 | Calamoceratidae (Trichoptera) | 7.7 | *Leiopotherapon unicolor* (spangled perch) | generalist predator |
| Barnett River | -0.2 | Calamoceratidae (Trichoptera) | 8.9 | *Hephaestus jenkinsi* (Jenkins grunter) | omnivore |
| Bayulu waterhole | 2.9 | Hydrophilidae (Coleoptera) | 9.0 | *Glossamia aprion* (mouth almighty) | generalist predator |
| Brooking Creek | 1.7 | Caenidae (Ephemeroptera) | 9.6 | *Neosilurus hyrtlii* (Hyrtls tandan) | benthivore |
| Fitzroy River (Bayulu) | 0.7 | zooplankton | 11.3 | *Strongylura krefftii* (longtom) | piscivore |
| Fitzroy River (Bluebush) | 2.9 | Simuliidae (Diptera) | 9.8 | *Glossogobius giuris* (flathead goby) | insectivore |
| Fitzroy River (Noonkanbah) | 1.5 | zooplankton | 11.5 | *Hephaestus jenkinsi* (Jenkins grunter) | omnivore |
| Fossil Downs waterhole | 3.8 | Naucoridae (Hemiptera) | 10.6 | *Hephaestus jenkinsi* (Jenkins grunter) | omnivore |
| Gillagoowa Spring | 0.8 | Gastropoda | 9.4 | *Anguilla bicolor* (Indian short-finned eel) | generalist predator |
| Hann River | 1.4 | Caenidae (Ephemeroptera) | 8.5 | *Leiopotherapon unicolor* (spangled perch) | generalist predator |
| Manning River | -0.3 | Helicopsychidae (Trichoptera) | 7.4 | *Mogurnda oligolepis* (gudgeon) | insectivore |
| Margaret River (Muludja) | 1.4 | Leptoceridae (Trichoptera) | 10.5 | *Hephaestus jenkinsi* (Jenkins grunter) | omnivore |
| Margaret River (Yiyili) | 2.7 | Caenidae (Ephemeroptera) | 9.4 | *Leiopotherapon unicolor* (spangled perch) | generalist predator |
| Mary River | 1.4 | Caenidae (Ephemeroptera) | 9.2 | *Hephaestus jenkinsi* (Jenkins grunter) | omnivore |
| Mt Pierre Creek (Galeru Gorge) | 0.5 | Chironomidae (Diptera) | 9.4 | *Leiopotherapon unicolor* (spangled perch) | generalist predator |
| Traine River | 1.4 | Leptophlebiidae (Ephemeroptera) | 9.0 | *Ambassis* sp. (glassfish) | invertivore |
| Troys Lagoon | 1.8 | Gastropoda | 8.7 | *Melanotaenia australis* (rainbowfish) | omnivore |
